# An application of social marketing for promoting HIV testing in Iran

**DOI:** 10.1186/s12889-023-15698-5

**Published:** 2023-05-11

**Authors:** Fatemeh Alipour, Mohsen Shams, Mostafa Maleki, Ali Mousavizadeh

**Affiliations:** 1 Department of Health Education and Promotion, School of Health, Yasuj University of Medical Sciences, Yasuj, Iran; 2 Department of Health Education and Promotion, School of Health, Tehran University of Medical Sciences, Tehran, Iran; 3 Department of Biostatistics and Epidemiology, School of Health, Social Determinants of Health Research Center, Yasuj University of Medical Sciences, Yasuj, Iran

**Keywords:** HIV/AIDS, HIV test, Social Marketing, Campaign

## Abstract

**Background:**

It has been estimated that 60,000 Iranians have been infected with HIV/AIDS and only 36% of them are aware of their status. This study aimed to design, implement and evaluate a social marketing campaign to promote HIV testing in Boyer-Ahmad County, Kohgiluyeh, and Boyer-Ahmad Province, southwest of Iran.

**Materials and methods:**

This study was a quasi-experimental pretest-posttest without a control group, developed based on a social marketing assessment and response tool. To design the intervention formative research was conducted, comprised of four focus group discussion sessions with 42 participants of the target community along with seven in-depth semi-structured personal interviews with health care providers involved in the HIV/AIDS Program. Data analysis was done manually using content analysis and the main content was formulated for the campaign. Afterward, the slogan and messages of the campaign were developed. The campaign’s materials including banners, posters, pamphlets, referral forms, and short messages were designed, pretested, and revised. Ultimately, the campaign was conducted for one month in October 2019. To determine the effectiveness of the campaign, the rate of referrals to the Center for Behavioral Health Counseling Services (CBHCS), for three months before and after the campaign, was compared.

**Results:**

Qualitative findings identified that the majority of the interviewees mentioned that the main reasons for the low rate of referrals to get tested for HIV were lack of awareness and information about HIV/AIDS and its diagnosis as well as the free and confidential tests available from the CBHCS. Moreover, the stigma associated with HIV/AIDS was another important reason for low referrals for testing. The rate of referrals for HIV testing in the three months leading up to the campaign was 18, 32, and 23 people, and three months after the campaign was 64, 81, and 44 individuals; respectively. The results of the multivariate analysis demonstrated that the campaign had increased the rates of referrals for HIV testing through its significant influence on females, and individuals with academic degrees.

**Conclusion:**

It can be concluded that the social marketing campaign was successful in persuading people to get tested for HIV.

## Introduction

As a worldwide emergency, the human immunodeficiency virus infection and acquired immune deficiency syndrome (HIV/AIDS) epidemic is one of the biggest challenges facing humanity [1]. HIV/AIDS is the fourth leading cause of mortality and the second largest infectious disease causing death worldwide [2]. There are 37.9 million people worldwide living with HIV/AIDS, including 1.7 million newly infected individuals and 770,000 patients, who lost their lives in the same year due to this condition [3]. The World Health Organization (WHO) has identified that the number of people suffering from HIV/AIDS in low-and middle-income countries (LMICs) is five times higher than the figures recorded [4] with over 90% of cases also occurring in LMICs [5]. Although, the number of deaths resulting from HIV/AIDS globally has dropped by 37% compared to 2005, deaths from HIV/AIDS have risen by 66% in the Eastern Mediterranean Region and North Africa including Iran, attributable to problems in the domains of diagnosis, care, and treatment of infected populations [6].

The status of HIV/AIDS in Iran has been described as a time bomb, whose detonation will lead to a growing number of infected individuals [7]. According to the evidence, 61,000 people are living with HIV/AIDS in Iran, including 4,400 newly infected cases [8]. Among individuals suffering from HIV/AIDS, only 36% of them are aware of their condition, 20% of the individuals are receiving treatments, and in 17% of cases, HIV virus load in their body has decreased [8]. Based on the information obtained from the office of the HIV/AIDS program in the Kohgiluyeh and Boyer-Ahmad Province, with a population of 713,052 people, 207 identified cases of HIV/AIDS, including 104 individuals who have died of the disease and 91 cases undergoing care. Also, it is estimated that there are 507 people living with HIV/AIDS in this province [9]. In other words, only 40% of the patients are aware of their condition. Boyer- Ahmad County with 299,885 inhabitants and the most populated city in the province, has only 20 individuals diagnosed with HIV/AIDS.

According to the Iran Ministry of Health and Medical Education, 50% of all cases at the time of diagnosis have been in the age group of 20 to 35 years, a pattern that has not varied over recent years [10]. Youth are considered the most vulnerable communities to HIV/AIDS infection as this group in also known to engage in a range of other high-risk health behaviors such as smoking, drug and alcohol abuse, and unsafe sex [11]. Various evidence reveals that about half of young Iranians have experienced at least one of these risky health behaviors [12, 13].

Testing is regarded as the first step in diagnosing HIV seropositivity and encouraging people testing positive to receive care and reduce possible modes of transmission. High-risk behaviors are likely to change as individuals learn about their infection status with anti-viral treatments able to effectively moderate the disease burden [14]. On account of the social stigma to HIV/AIDS in Iranian society, most young people and vulnerable communities do not refer to healthcare or specific centers to receive counseling services and education on how to diminish the impact of high-risk behaviors [15]. On the other hand, unknown infections can be one of the factors contributing to HIV/AIDS transmission among different populations [16]. If HIV testing can identify HIV positive cases, then HIV transmission to sexual partners and children can be reduced and patients can also receive treatment [17]. Therefore, reducing stigma and promoting early HIV testing is very important.

However, within the Iranian health system, the evidence points to a greater emphasis being placed on designing specialist-oriented educational programs rather than elevating the opinions and views of those in the HIV/AIDS target communities when designing such programs [18]. While in many countries, audience-oriented approaches have been used to design interventions to promote HIV testing [19]. Many of these interventions are designed based on social marketing approaches [20]. Social marketing takes account of the desires and opinions of target groups by using appropriate research methods in the design of interventions to increase the benefits of behavior, reduce barriers, or increase motivations toward desirable behaviours [21]. Accordingly, the present study was conducted to design, implement and evaluate an evidence based, best practice, social marketing campaign to promote HIV testing in Boyer-Ahmad County, Kohgiluyeh and Boyer-Ahmad Province, southwest of Iran.

## Materials and methods

### Social marketing assessment and response tool (SMART) model

The SMART model was used in this study, whose phases are preliminary planning; audience, channel, and market analysis, educational materials development, pretesting, implementation, and evaluation [22]. The preliminary planning phase was done by identifying the problem and reviewing the resources available to the research team. Social marketing programs are dominantly based on conducting formative research to design interventions to ensure proper responses to the demands and needs of the target audience [23]. The formative research consists of audience analysis, market analysis, and channel analysis, conducted in this study in the form of a qualitative study (focus group discussions and in-depth semi-structured interviews). The views and comments of the target group as well as experts involved in HIV/AIDS programs were identified during this formative study. Issues included promoting of HIV testing, elements of behavioral marketing, appropriate and effective communication channels and initial ideas for the interventions.

### Study design and setting

This study used a quasi-experimental pretest-posttest design without a control group. The study comprised a qualitative study to identify the intervention content and structure, implementation, and evaluation phases. The study was carried out in Boyer-Ahmad County, southwest of Iran, in 2019.

### Qualitative study

The qualitative study was conducted based on the social marketing approach. It aimed to obtain the opinions of the interviewees as to why people of Boyer-Ahmad County do not go for HIV testing, obstacles that make them less likely to go for HIV testing, appropriate messages and methods to convince people to get tested for HIV, and effective communication channels and suitable places to convey campaign messages to the people.

The qualitative data was collected through focus group discussions (FGD) with members of the target community and in-depth semi-structured interviews with healthcare providers involved in the HIV/AIDS program.

For the FGD, the participants were purposefully selected and divided into four groups segmented by age (21–35 years old), sex (male and female), level of education (having or not having a university degree, and place of residence (rural and urban). The number of individuals in each group was 8–12 people. It should be noted that the reason for selecting the participants from the age group of 21–35 years was that 51% of HIV/AIDS patients are in this age group [9].

Participants were recruited for FGDs using information from the SIB-Integrated Health Record System available from the Akbarabad and Mehriyan Healthcare Center. Prior to the commencement of each FGD session, informed consent was obtained from all participants and participants were assured that their information would remain confidential. Inclusion criteria for the FGDs included people aged 21 to 35 living in Boyer-Ahmad county who had informed written consent to participate in the study. Exclusion criteria was unwillingness to continue to participate in the study for any reason.

A female moderator facilitated all the groups with four FGDs conducted with 42 members of the target community. Further FGDs were not conducted due to data saturation and duplication of opinions and content. Each FGD session lasted about 90 min. At the end of the sessions, the conversations recorded by the researcher were also transcribed and then compared with the notes taken. Ultimately, the final draft of the transcriptions was formulated and the data-set was prepared for content analysis.

The following semi-structured questions were used to collect qualitative data for the FGDs. Questions were designed according to the aims of the study and the principles of formative research in the social marketing approach, including the following questions: What do you think is important about early detection of HIV infection? What do you think are the reasons for low referrals to HIV test? If people want to get tested for HIV, where are the right places? When is the best time for people to come in for HIV test? What channels can be used to encourage people to get tested for HIV? What can be done to persuade people to get tested for HIV?

In-depth semi-structured personal interviews with seven healthcare providers involved in the HIV/AIDS Program were another aspect of the qualitative study. To do this, participants were visited in their workplaces, explained the study objectives, and invited for an interviews with times and locations for the interviews established. Each interview took an average of 60 minutes. Signed consent was requested prior to interviews commencing with all interviews also recorded. Recordings were then transcribed and the final draft was prepared for manual qualitative content analysis. Semi-structured questions of the in-depth interviews were as follows: What do you think are the reasons for the low number of people coming for an HIV test in Boyer-Ahmad county? In your opinion, what are the obstacles for the residents of Boyer-Ahmad city to go for an HIV test?

What do you think are the best communication channels with people to encourage them to get tested for HIV? What are your suggested ways to persuade people to come in for an HIV test?

Qualitative data were analyzed manually through content analysis using a concurrent analysis approach. The text files were carefully read several times by the researcher to gain a general understanding of the issues, and the sentences that answered the questions were identified. Then in each of these sentences, the main concepts were given a theme. In the next step, these themes were compared with each other to determine the main themes and sub-themes. Then the main themes and sub-themes were carefully read several times so that the main themes with similar meanings were placed in a category and formed classes.

### Intervention phase

Based on the qualitative results, the main content and structure were developed for the campaign. Based on the findings of the qualitative study the slogan for the campaign, key messages, and the final media materials were designed. Message pre-testing involved the messages and media materials given separately to a media specialist, a health education specialist, and an HIV program expert. Each of these experts provided their opinions to the research team. Based on the opinions of these experts, messages and media were modified and finalized. “Test, the only method to diagnose HIV”, was defined as a slogan, and “HIV test; fast, free, and confidential with professional Consultation; test now” developed as the key campaign message. Messages were then applied to campaign media materials such as banners, posters, pamphlets, referral forms, with other short messages designed, pretested, and reformed.

The social marketing interventions focused on a community-based campaign and the recipients of the services of the Behavioral Diseases Counseling Center were the main target group of the campaign. This center provides various health services to the whole community, one of these services is HIV testing. People who refer to this center do not have much desire to use services related to HIV/AIDS such as HIV testing.

The campaign was implemented in Boyer-Ahmad County for one month in October 2019. The components of the campaign included the following: Composing and sending SMS: At this stage, according to the qualitative results, the content of the SMS was composed, pretested, and revised by the research team and target group, and then it was sent to 15,625 individuals of the target community via the Telecommunication Company of Iran (TCI). The content of the SMS included the slogan, the message of the campaign, and the address of the HIV testing health facility center.

Other resources for the intervention stage included the design and installation of 10 banners in the healthcare centers at Boyer-Ahmad County, the campus of Yasuj University of Medical Sciences, and dental clinics, the installation of 100 posters and 300 pamphlets at the target community’s gathering places dormitories, offices, and dental clinics and also provision of electronic copies of resources to key officials universities, student associations, university unions, and public relations departments. Other components of the intervention were: Posting campaign messages on social media of the target group and provincial websites, developing and submitting the newsletter entitled “Promotion of HIV testing” to local news websites of Boyer-Ahmad Country, distributing the referral forms in the Family Health Unit of Shahid Ashrafi Healthcare Center, responsible for holding pre-marriage counseling programs.

To determine the effectiveness of the campaign, the number of people referred to the Behavioral Diseases Counseling Center for HIV testing three months before the campaign and two months after the campaign were compared. The province’s comprehensive electronic HIV/AIDS data management system was used to determine the number of people who were referred to the Behavioral Diseases Counseling Center for HIV testing. The Chi-square test and multivariate analysis were used for quantitative data analysis. The Chi-square test was used to find a significant association. All significant variables were then included in a multivariate analysis. SPSS Version 21 was used to analyze the data.

## Results

### Formative research findings

The formative research included FGD sessions and in-depth semi-structured personal interviews. The FGDs included 42 members of the target community with a mean and standard deviation (SD) age of 28.5 ± 3.53 years old. As well, 38% of participants were male (16) and 62% were female (26). Moreover, 55% of participants had academic degrees (23) and 45% had lower than high school diplomas (19).

Findings from the formative research revealed that high level of stigma in the community towards HIV/AIDS and its transmission, low level of public awareness about the disease and its transmission, and no knowledge about the CBHCS including free and confidential rapid diagnostic tests were the main reasons of low rates of referrals for HIV testing. The fear of positive test results and no notification by healthcare centers were other reasons mentioned. The participants also introduced cyberspace and the IRIB as the best communication channels to convey messages. Furthermore, they stated that the best way to encourage people to do the tests was giving them awareness about the disease and its transmission, testing time and place, free and confidential tests, as well as SMS with effective content. The intervention was designed according to these results. The summary of the results of the qualitative study is presented in Table 1.

**Table 1:** Summary of the findings of the qualitative study

Main themes	Findings
Important of early detection of infection	Prevent transmission to others Prevent from the disease entering advanced stages More motivation for treatment
Barriers to HIV testing	Society’s negative view of the disease and its ways of transmission Low level of public awareness about the disease and its symptoms Low level of public awareness about the ways of transmission, diagnosis, and treatment Fear of the name of the disease (terrible name of the disease) Fear of a positive test result Fear of looking bad at others and being rejected by family and society Personal problems: Lack of time, doing housework Laziness and carelessness about your health Great importance to people’s words Lack of advice and information by health centers and responsible institutions Ensure being healthy Misconceptions about the disease: It is better not to know what disease we have
Proper places for HIV testing	Prior information and testing in public places such as parks Prior information and testing in public places such as parks Prior information and testing in universities and schools Prior notification and testing in laboratories Prior notification and testing in addiction treatment camps Prior notice and testing along with marriage testing Prior notification and testing in military barracks, hospitals, blood transfusion centers
Proper communication channels for disseminating messages	Cyberspace: Telegram and WhatsApp groups Broadcast: Dena Nights Program, News SMS system: Collecting phone numbers by Behvarz and sending SMS Print media: Banners in the city and parks Parent-teacher meetings in schools Influential people: trusted people of the place, Behvarz, mother of the family Friday prayer sermons
Ways to persuasion people	Information about the disease, place, and time of the test Inform each area through the health house and perform tests Information about the free and confidentiality of the test Include other tests with AIDS testing Intimidation and coercion Normalization; It is constantly mentioned in the media SMS with impressive text Advertise through people who have experimented Accuracy of experiments Donate a health card to clients after the test The correct behavior of the staff with the clients Continuous advertising Perform tests in several places so that people do not procrastinate Distribute small advertisements of the program among the routine clients of health centers Clarification about the disease Talking to families and at-risk groups, including addicts Have an AIDS test card like an organ donation card

### Post-intervention findings

Two months after the campaign, data was extracted from the country’s comprehensive electronic HIV/AIDS data system relating to the monthly referral status to the center for HIV testing. In the three months before the campaign, the mean and SD of the age of the 73 people referred for an HIV test was 30.39 ± 8.27, and two months after the campaign the mean and SD of the age of the 188 people referred to do HIV test was 32.44 ± 11.11.

**Fig. 1:**
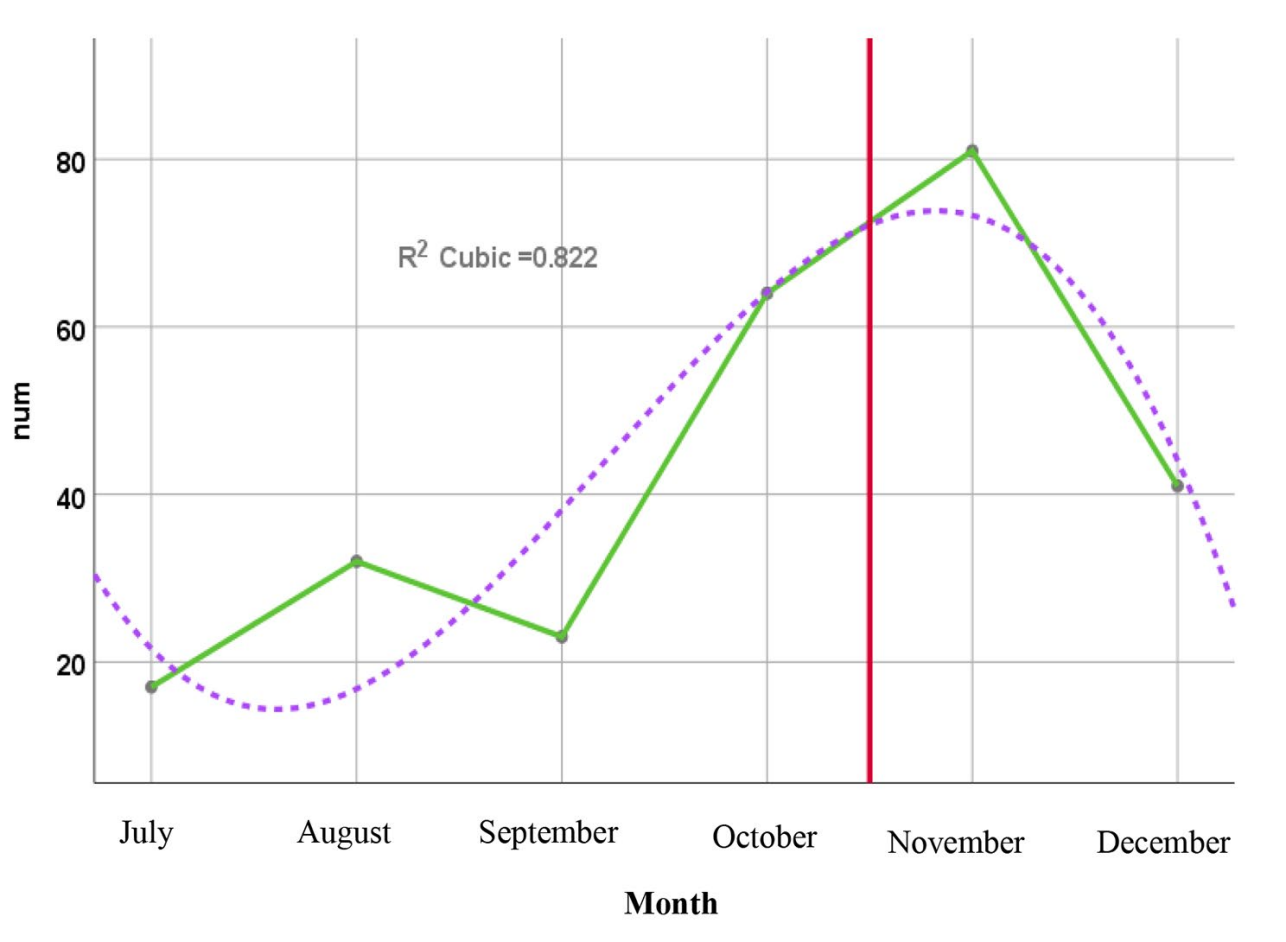
Status diagram of referrals to the CBHCS for HIV testing before and after the campaign

Referrals to the CBHCS during the three months before the campaign i.e. July, August, and September were 18, 32, and 23 cases, respectively. But, in October when the campaign was implemented, the testing rate rose by 64 people and it was 81 and 44 cases in November and December; respectively (Fig. 1). The peak of referrals to the center occurred one month after the campaign.

Three months before the campaign, 27.8% of the total referrals rate to do HIV test was female, but this figure increased significantly by 35.1 after the intervention. In terms of education, 40.3% of the people to do HIV test had academic degrees before the campaign and this rate reached 66.7% after the campaign. This increase was statistically significant. Before the campaign, 30.6% of the referrals included individuals living in urban areas and this value increased significantly by 50% after the campaign (Table 2). Moreover, the results of the multivariate analysis demonstrated that the campaign had increased the rates of referrals for HIV testing through its influence on the female (P-value = 0.01, Confident interval 95%= 0.29–2.61) and individuals with academic degrees (P-value = 0.02, Confident interval 95%= 0.02–0.29).

**Table 2:** The rate of referrals for HIV testing before and after the campaign based on demographic variables

Demographic variables		Before the intervention (Percent) Number	After the intervention (Percent) Number	P-Value
Sex	male	(72.2) 52	(64.9) 12	df = 1, Chi square = 1.26 P-Value = 0.04
	female	(27/8) 20	(35.1) 66	
Degree of education	Nonacademic	(59/7) 43	(33.3) 62	df = 1, Chi square = 15.51 P-Value = 0.004
	Academic	(40.3) 29	(66.7) 124	
Place of living	Rural	(27.8) 20	(12.8) 24	df = 2, Chi square = 11.61 P-Value = 0.003
	Urban	(30.6) 22	(50) 94	
	No mentioned	(41.7) 30	(37.2) 70	

The time trend diagram showed a change in the number of referrals to the center based on a third-order function pattern and the peak occurring one month after the campaign. The return of the referral trend to lower levels in the final month of the campaign might either indicate the necessity for the continuation of the campaign.

## Discussion

Although some high income countries have made significant achievements in controlling the HIV/AIDS epidemic, this condition is expanding in LMICs [24]. It is thus a serious issue drawing the attention of researchers and healthcare providers. Despite efforts made to increase rates of referrals for testing and remove barriers, the number of Iranian people referring to get tested for HIV has been low [6]. Therefore, this intervention was designed, implemented, and evaluated to promote HIV testing in Boyer-Ahmad County based on the social marketing approach.

Based on the findings of the qualitative study, a set of factors play a role in the low referral of people for HIV testing. The negative view of society toward the disease and the ways of its transmission and the stigma associated with the disease were one of these reasons. This finding is in line with the results of a study conducted in the port of Cape Town in Africa. In this study, the stigma of the disease was significantly higher among people who had not tested for HIV than among people who had tested [25].

Fear of a positive HIV test result, fear of being rejected by the family and community in case of being infected, and low perceived susceptibility were mentioned as other obstacles. These barriers have also been stated in similar studies [26–28]. In a study in China, fear of a positive test result and fear of discrimination in case of a positive test, and low perceived risk of the disease were reported as the main factors of low referral of men having sex with men for HIV [26] testing.

In another study aimed at investigating the barriers to HIV testing in Europe, it was found that low perceived risk of the disease, fear, and worry of being infected, and fear of disclosing patient information were raised as barriers to testing [27]. In a qualitative study to study the factors affecting the non-participation of pregnant women in the program to the prevention of mother-to-child HIV transmission, fear of knowing their HIV status, the stigma associated with the infection, lack of support from their partners, and negative attitudes toward health workers were reported as obstacles to HIV testing [28].

Based on the results of the qualitative research of the current study, social networks and IRIB were proposed as the best channels for disseminating HIV testing promotion messages. In line with the results of the present study, the use of virtual space and mass media has been reported as appropriate and effective channels [29–31].

The interviewees in the qualitative study offered the following suggestions to encourage people to get tested for HIV: Increasing community awareness about HIV, informing about the place and time of the HIV test, as well as the free and confidential nature of the test, conducting the HIV test simultaneously with other tests, sending SMS to the community members with effective and persuasive content, and the proper and effective communication of the health care provided with the clients, intimidation, and coercion. These results except resort to intimidation and coercion are consistent with the findings of the Blas study. In this study, it is reported that interventions should be designed based on motivational messages to persuade participants to perform the test. The intervention messages should increase the perceived risk of HIV. Also, the messages should emphasize the confidentiality of the test result, the professionalism of the counseling and testing staff, the complete explanation of the HIV testing process, and the follow-up steps after receiving the test results. The messages should also contain information about the location and time of the test and the personnel providing the test services [32]. Although the interviewees suggested resorting to intimidation and coercion as a method to increase people’s referrals for HIV testing, intimidation and coercion are neither ethical nor effective. Therefore, this suggestion was ignored in the design of the intervention.

The data after the intervention showed that the campaign was successful in increasing the number of referrals for HIV testing. This change was significant in individuals with academic degrees, and residents in urban areas compared with those at the pre-intervention stage. Social marketing campaigns have been conducted around the world, especially in high-income countries, to promote HIV testing [16, 20, 33–35]. Some of these campaigns have been successful in increasing HIV testing and some have not. For example, following a social marketing campaign in Canada, HIV testing among the target group increased by 23% [35]. In the “Make Your Position Clear” campaign in Scotland, the intention to get tested for HIV in the next six months increased significantly [36]. In The Saving Lives campaign in England, 16% of target group members took an HIV test as a result of exposure to the campaign [37]. In contrast to these campaigns, there was no significant change in the number of HIV tests during or after the “Check It Out” campaign in Australia and the “I Did It” and “Count Me” campaigns in England [38, 39].

Other findings of this study showed that people with a better educational status and urban area residents were significantly more affected by the campaign and took an HIV test. Other studies also show that educational status and living in urban areas are significant determinants of HIV testing [40–42]. In the study with the target group of 15 to 49 years old, it was found that the probability of voluntary testing is higher among people with higher education and city residents [43]. As the evidence shows, it seems that more knowledge about HIV/AIDS makes people more willing to get tested for HIV. The other reasons for the acceptance of HIV testing by people with better educational status can be wider information in academic environments, and receiving more campaign messages by the student population due to their greater use of social networks. It has been found that people with a lower level of education have less health literacy [44, 45] and low level of health literacy is linked to the low level of referral for HIV [46, 47]. Accordingly, perhaps another reason for more acceptance of the test among people with higher education can be the better health literacy status among these people.

In addition, living in urban areas is also one of the determining factors in this regard. People living in the city have more and easier access to testing centers, and this can encourage them to get tested.

The most important limitation of the present study was the simultaneous event that happened in Chenar Mahmoudi village in Lordegan city. Due to the use of a common syringe for blood sampling, a number of people in the village were infected with HIV. This event caused an HIV epidemic among the villagers and led to sensitivity in society and mistrust among the people for HIV testing.

Setting up mobile teams to access high-risk areas and developing programs such as mobile HIV/AIDS buses were suggested as ways to improve access to the HIV test. But due to the HIV/AIDS epidemic in Lordegan County and the lack of rapid test kits as the barriers to the implementation of the program, they were excluded from the intervention. The data analysis was done using manual methods rather than qualitative software which may be perceived as a limitation. To determine the effectiveness of the campaign we compared the number of people referred for HIV testing before and following the interventions. However, an alternative approach of a pre-post KAP survey could be conducted to identify KAP determinants which may have contributed to motivating participants to attend the HIV testing facility.

## Conclusions

Based on the results of the study, it can be concluded that to increase people’s visits for HIV testing, it is necessary to first identify the existing barriers and reduce the effects of these barriers by designing effective interventions so that people are encouraged to voluntarily go for HIV testing. The social marketing approach provides a more comprehensive perspective to identify barriers and benefits to testing, and develop targeted and tailored messages delivered through appropriate channels of communication to increase opportunities for testing. As such, it can be concluded that despite the study limitations, the social marketing campaign was successful in persuading more people to get tested for HIV.

## Data Availability

The datasets used and analyzed during the current study are available from the corresponding author upon reasonable request.
